# High-throughput chromatin accessibility profiling at single-cell resolution

**DOI:** 10.1038/s41467-018-05887-x

**Published:** 2018-09-07

**Authors:** Anja Mezger, Sandy Klemm, Ishminder Mann, Kara Brower, Alain Mir, Magnolia Bostick, Andrew Farmer, Polly Fordyce, Sten Linnarsson, William Greenleaf

**Affiliations:** 1 Department of Genetics, Stanford University, Stanford, CA 94305 USA; 2 Department of Medical Biochemistry and Biophysics, Karolinska Institute, Stockholm, 17177 Sweden; 3 Takara Bio USA, Mountain View, CA 94043 USA; 4 Department of Bioengineering, Stanford University, Stanford, CA 94305 USA; 5 ChEM-H Institute, Stanford University, Stanford, CA 94305 USA; 6 Chan Zuckerberg Biohub, San Francisco, CA 94158 USA; 7 Department of Applied Physics, Stanford University, Stanford, CA 94305 USA

## Abstract

Here we develop a high-throughput single-cell ATAC-seq (assay for transposition of accessible chromatin) method to measure physical access to DNA in whole cells. Our approach integrates fluorescence imaging and addressable reagent deposition across a massively parallel (5184) nano-well array, yielding a nearly 20-fold improvement in throughput (up to ~1800 cells/chip, 4–5 h on-chip processing time) and library preparation cost (~81¢ per cell) compared to prior microfluidic implementations. We apply this method to measure regulatory variation in peripheral blood mononuclear cells (PBMCs) and show robust, de novo clustering of single cells by hematopoietic cell type.

## Abstract (web summary)

Single-cell chromatin accessibility is a promising means to identify regulatory programs in mixtures of cells. Here the authors describe µ-ATAC-seq, a low-cost method that can generate thousands of accessibility profiles per day.

## Introduction

A central challenge of systems biology is to determine the epigenome of phenotypically distinct cellular states within complex primary tissue. Toward this goal, single-cell chromatin accessibility measurements provide an important epigenetic view of the regulatory landscape within individual cells by capturing the physical accessibility of putative functional elements across the genome^1–6^. Methods for measuring chromatin accessibility at single-cell resolution, however, are low throughput, depth limited, or require complex molecular processing to generate cellular indexing reagents^2–5,7^. For ultra-high throughput accessibility profiling applications, combinatorial indexing approaches^2,7^ offer significant promise, yet these methods capture fewer accessible fragments per cell than single-cell isolation technologies^1,3^ and are not amenable to integration with single-cell microscopy or other multi-omic assays that require whole, live cells. In this report, we describe a high-throughput implementation of single-cell ATAC-seq^8^ (scATAC-seq) that directly integrates fluorescence imaging and provides an extensible foundation for multi-omic epigenetic profiling in single cells.

## Results

### Implementation of scATAC-seq on nanoliter-scale wells

**Fig. 1:**
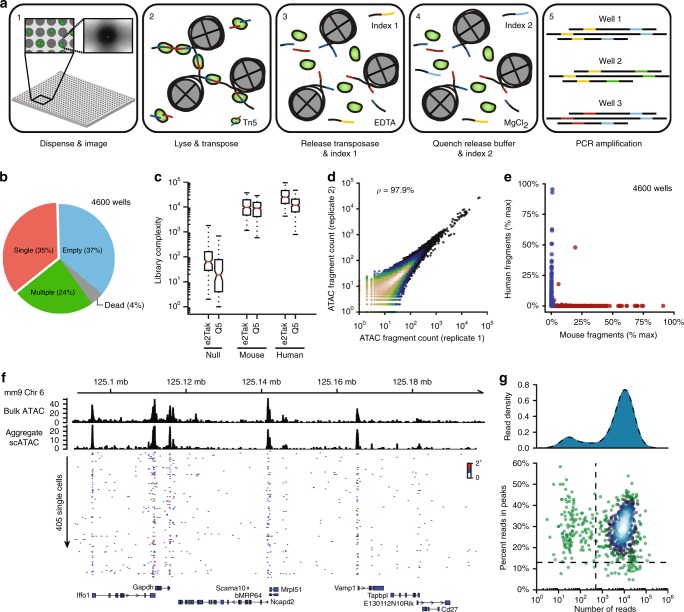
µATAC-seq: a nano-well scATAC-seq implementation on the ICELL8 platform. **a** µATAC-seq workflow. **b** Distribution of cell counts per well measured by fluorescence microscopy (Hoechst). **c** µATAC-seq library complexity for null, mouse, and human targeted wells using two separate polymerases (e2Tak and Q5) for well barcoding and amplification (*n* = 5000 wells). For each sample, the box denotes the interquartile range centered at the median (red line), while the whiskers span the 5th and 95th percentile range. **d** Correlation between nano-well chips processed with either a e2Tak (replicate 1) or Q5 polymerase (replicate 2) across all accessible loci. **e** Inter-well mixing of mouse and human µATAC-seq fragments. **f** Representative population^22^ and single-cell ATAC-seq genome tracks for the Gapdh locus. **g** Signal-to-background (percent reads in peaks) as a function of read depth (*n* = 792). Only cells lying in the upper right quadrant (marked by dashed lines) are retained for downstream analysis

We have implemented scATAC-seq in small volumes (µATAC-seq) using a recently developed nanoliter-scale liquid deposition system (ICELL8 Single Cell System, Takara Bio USA). This approach reduces reagent costs and achieves equal or higher per-cell fragment counts than prior state-of-the-art implementations^2,3,7^. The workflow—illustrated in Figure 1a—is comprising of the following steps: (1) isolated single cells are stained with Hoechst and propidium iodide and stochastically loaded under Poisson statistics (~1 cell per well on average) across 5184 wells under active humidity and temperature control; all wells are then imaged via multi-color microscopy to identify those containing a single-live cell; (2) transposition reagents are added to a selected set of wells (e.g., those containing a single live cell) and incubated at 37 °C for 30 min; (3) the transposition reaction is quenched by incubation with EDTA; (4) MgCl_2_ is added in equimolar concentration to quench the chelating capacity of EDTA in preparation for subsequent PCR amplification; (5) PCR reagents are added and µATAC-seq fragment libraries are amplified using barcoded primers provided in the prior two steps (see Supplementary Table 1 for reagent loading chart). Following on-chip library construction, indexed µATAC-seq libraries are extracted from all nano-wells by centrifugation, purified, and then further amplified as necessary for sequencing (Methods section).

### Benchmarking analysis of µATAC

As an initial test of µATAC-seq, we loaded samples into 5000 wells across two nano-well ICELL8 chips. On each chip, 200 wells were loaded with PBS (designated null wells); 1150 wells were loaded with mouse embryonic stem cells (mESCs, ~1 cell per well); and 1150 wells loaded with human lymphoblastoid GM12878 cells (~1 cell per well). This yielded a total of 4600 wells targeted with either human or mouse cells across both chips. Imaging of Hoechst and propidium iodide fluorescence revealed the anticipated fraction of wells containing live single cells (35%, 1616 single cells), consistent with near optimal loading that maximizes the number of single-cell containing wells (Fig. 1b). Barcoded sequencing of each of the 5000 targeted wells revealed 14.3 × 10^3^ (8.1 × 10^3^) median fragments per single human (mouse) cell containing wells (*n* = 1616)—reflecting a two orders of magnitude enrichment over null wells (Fig. 1c and Supplementary Figure 1a,b). These library complexities compare favorably with microfluidic cell capture (5.8 × 10^3^ fragments per GM12878 cell^3^) as well as combinatorial indexing (2.5 × 10^3^ fragments per GM12878 cell^7^) approaches. The µATAC-seq libraries capture both sub-nucleosome as well as nucleosome length fragments, yet, the median fragment length is shorter than that observed using the Fluidigm C1 platform. Consistent with prior bulk and single-cell ATAC-seq libraries, we observe a more than tenfold enrichment for fragments proximal to transcription start sites (TSS) relative to distal regions, reflecting a high fraction of fragments captured within open rather than closed chromatin (Supplementary Figure 2a). Furthermore, we find a high degree of concordance (97.9%) between nano-well chips even when µATAC-seq fragments are amplified with different polymerases (Fig. 1d). We further tested the deposition fidelity of the ICELL8 platform, observing both human and mouse cells in fewer than 0.2% of wells (Fig. 1e).

Aggregate single-cell profiles recapitulate population measurements broadly across the accessible genome (Supplementary Figure 2b) as well as specifically at individual genomic loci (Fig. 1f). At single-cell resolution, accessibility profiles are enriched for open chromatin (Fig. 1f, g) in both mESCs (29% reads in peaks, Fig. 1g) and GM12878 cells (22% reads in peaks, Supplementary Figure 2c). Collectively, these data establish the proposed nano-well implementation as a high-throughput framework for scATAC-seq library construction.

### Epigenetic signature distinguishes PBMC types

**Fig. 2:**
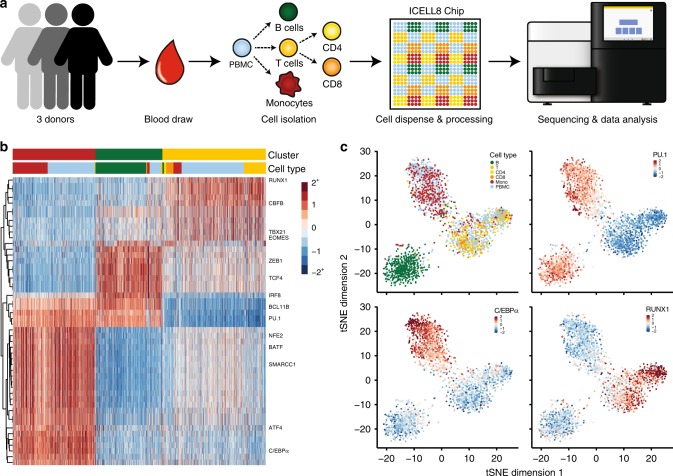
De novo identification of hematopoietic cell types by µATAC-seq. **a** Human PBMC isolation and µATAC-seq workflow. **b** Hierarchical (TF motifs) and k-means (cells) clustering of accessibility deviation *z*-scores across 2333 single cells (columns) of the 50 most variable TF motifs (rows). Colors correspond to cell types defined in **a**. **c** tSNE visualization of accessibility deviations at TF motifs for cells in **b**. Cells are either colored by cell type (upper left panel) or by the accessibility deviation *z*-score for the specified TF motif

We next asked whether µATAC-seq epigenetic profiles are sufficient to distinguish cell types within complex primary tissue. For this purpose, we performed µATAC-seq on human peripheral blood mononuclear cells (PBMCs) as well as B, T, CD4^+^ T, CD8^+^ T, and monocyte cells isolated directly from whole blood (Fig. 2a), yielding 2333 single cells passing all quality control criteria (Methods section). Using ChromVar, a bioinformatic approach described previously^9^, we calculated the relative accessibility of transcription factor (TF) binding motifs in individual cells and found that isolated B, T, and monocyte cells robustly cluster by cell type (Supplementary Figure 3a). By aggregating fragments within single cells that are proximal to a TF motif, this epigenetic signature captures the variation in putative TF binding site accessibility across a population of cells^9^. A relatively small fraction of cells are incorrectly assigned to clusters; however, the frequency of these events as well as the random distribution of these cells within apposing clusters both suggest that isolation impurity upstream of the µATAC-seq assay is the primary source of these errors (Supplementary Table 2). PBMC subpopulations co-cluster precisely with the isolated cell types (Fig. 2b, c), showing highly concordant cell type-specific accessibility patterns within appropriate tSNE^10^ (t-Distributed Stochastic Neighbor Embedding) clusters (Fig. 2c) as well as k-means clustering across highly variable TF binding motif accessibility patterns (Fig. 2b). Consistent with published gene expression data, we find that the PU.1 binding motif is differentially accessible in monocytes and B cells relative to T cells (Fig. 2c, upper right panel)^11,12^, the C/EBPα motif is exclusively accessible in monocytes (Fig. 2c, lower left panel)^13,14^, and RUNX1 motif accessibility is appropriately enriched in T cells—reflecting the broad regulatory role of the RUNX protein family in T lymphocytes (Fig. 2c, lower right panel)^15^. These results are highly robust to biological (three human blood donors) and technical variation (Supplementary Figure 3b). To further establish the robustness of clustering by cell type, we independently purified CD4^+^ and CD8^+^ T cells and found that these subtypes co-cluster with independently isolated T cells (Fig. 2c, upper left panel). Collectively, these data suggest that µATAC-seq signatures are sufficient for de novo clustering of PBMCs by hematopoietic cell type.

## Discussion

In this report, we have described µATAC-seq—a high-throughput, single-cell chromatin accessibility assay that dramatically reduces per-cell costs, requires only commercially available reagents, provides state-of-the-art data quality, and increases throughput nearly 20-fold over existing single-cell capture technologies. Single-cell chromatin measurements present a unique experimental challenge since only two DNA templates are present in a diploid cell. Technical sampling noise as well as biological heterogeneity further confound this problem, resulting in a ~10% observation efficiency of accessible regions in single cells^3^. Consequently, a few hundred cells are typically required to reliably determine the accessibility landscape of each subpopulation within a mixture of cells. Our approach in this work has been to develop an experimental framework for processing more than a thousand of cells in parallel to determine the accessibility of multiple cell types within a complex tissue. In general, nano-well single-cell sequencing approaches such as µATAC-seq are highly extensible, well-suited for multi-omic analysis, and define an important direction for single-cell epigenetic methods development.

## Methods

### Cell culture

All cell lines were grown at 37 °C with 5% CO_2_. GM12878 cells were obtained from the laboratory of Michael Synder (Stanford Univeristy) and were cultured in RPMI 1640 media supplemented with l-glutamine (Thermo Fisher Scientific, MA, USA, Cat. #11875–085) and 10% FBS (Thermo Fisher Scientific, Cat. #10082147); mESC cells (129S1X Castaneous, gift from Howard Chang) were cultured in 15% FBS (HyClone GE Healthcare Life Sciences, SH30070.03E) supplemented with non-essential amino acids, l-glutamine and Leukemia Inhibitory Factor (LIF, Invitrogen, Cat. #A35935). Adherent mESCs were washed twice in 1X PBS and detached using trypsin (Sigma, MO, USA) for 5 min. Cells were diluted in their respective media, collected by centrifugation at 400 g for 5 min, and then resuspended in media.

### Immune cell isolation from whole blood

Monocytes, T cells, CD4^+^ T cell, CD8^+^ T cells, and B cells were isolated from whole blood (AllCells, CA, USA) using EasySep Direct Human cell isolation kits (STEMCELL Technologies, MA, USA) according to the manufacturer’s protocol. Isolated PBMCs (AllCells, CA, USA) were thawed in RPMI and washed once in media before staining the cells as described below. All human cells were obtained from AllCells with explicit consent to publish data for broad genomic release.

### ICELL8 workflow

Cells were stained with Hoechst and propidium iodide using the ReadyProbes Cell Viability Imaging Kit (Thermo Fisher Scientific) for 20 min in media at 37 °C, then washed twice in cold 0.5X PBS. Cells were counted and dispensed into nano-wells using the SMARTer^TM^ ICELL8^**®**^ Single-Cell System (Takara Bio USA, CA, USA, Cat. #640000) at 25 cells/µl in 0.5X PBS, 1X Second Diluent (Takara Bio USA, Cat. # 640196) and 0.4 U/µl RNase Inhibitor (New England Biolabs [NEB], MA, USA) into a SMARTer ICELL8 250v chip (Takara Bio USA, Cat. #640183). Control wells containing 1X PBS (25 µl) and fiducial mix (25 µl) (Takara Bio USA, Cat. #640196) were included in the source loading plate (see source plate loading chart in Supplementary Table 1). The on-chip deposition volume was 40 nl for all reagent delivery steps. The chips were maintained at 16 °C or lower between all reagent loading steps. Following cell deposition, chips were sealed with SMARTer ICELL8 imaging film (Takara Bio USA, Cat. #640014) and centrifuged at 400 g for 5 min at 4 °C and imaged with a 4× objective using Hoechst and propidium iodide fluorescence. Images were analyzed using automated microscopy image analysis software (CellSelect, Takara Bio USA). Immediately following imaging, the Tn5 transposition mix (2X TD buffer [20% dimethylformamide, 20 mM Tris-HCl, pH 7.6, 10 mM MgCl_2_], 100 µl Tn5 transposase [Nextera DNA Library Prep Kit, Illumina, CA, USA] per ml Tn5 transposition mix, 0.2% Tween 20, 0.2% NP40, and 0.02% Digitonin [Promega, WI, USA]) was dispensed. Chips were then sealed with imaging film, centrifuged at ~3000 g for 5 min at 4 °C and incubated for 30 min at 37 °C. To index the whole chip, 72 i5 and 72 i7 previously published, custom indices (Supplementary Table 3)^3^ were dispensed at 6.25 µM concentration with EDTA and MgCl_2_, respectively. To release the bound Tn5 transposase, 60 mM EDTA was dispensed together with the i5 indexes. After sealing the chip, it was centrifuged at 3000 g for 3 min and incubated for 30 min at 50 °C. Prior to performing PCR on-chip, the chelating capacity of EDTA was suppressed by dispensing 60 mM MgCl_2_ together with the i7 indices. Chips were then sealed with imaging film, centrifuged, and incubated at room temperature for 5 min. Finally, a PCR mix (5x Q5 [NEB] or e2TAK [Takara Bio USA] reaction buffer, 1 mM dNTPs [Thermo Fisher Scientific], and 100 U/ml Q5 [NEB] or 50 U/ml e2TAK polymerase [Takara Bio USA], respectively) was dispensed and 14 cycles of PCR were performed on-chip after sealing with TE Sealing film (Takara Bio USA, Cat. #640109) and centrifuging at ~3000 g (3 min) as follows: 5 min at 72 °C and 30 s at 98 °C followed by 14 cycles of 10 sec at 98 °C and 90 s (Q5 polymerase) or 150 s (e2TAK polymerase) at 72 °C, with a final extension of 2 min at 72 °C. PCR products were extracted by centrifugation at ~3000 g for 10 min using the supplied SMARTer ICELL8 Collection Kit (Takara Bio USA). All dispense and sealing steps were followed by centrifugation at ~3000 g for 3 min. All on-chip thermal cycling was performed using a SMARTer ICELL8 Thermal Cycler (Takara Bio USA).

### Off-chip purification and additional amplification

The collected PCR product was purified using MinElute PCR purification columns (Qiagen, Germany) following the manufacturer’s instructions. Due to the large sample volume, the PCR product was split across four MinElute columns, eluted in 10 µl volumes, and subsequently pooled. To remove free PCR primers, which would induce index-swapping during additional rounds of off-chip amplification, we performed two rounds of bead clean-up using Ampure XP beads (Beckman Coulter, CA, USA) in a 1:1.2 ratio. The beads were incubated for 8 min with the PCR product, washed twice in 70% ethanol, and eluted in 20 µl ultrapure water (Thermo Fisher Scientific). Further amplification was required only for the mouse and human mixing experiment. PBMCs libraries generated on-chip were directly sequenced following column and bead purifications.

The number of required off-chip amplification cycles was determined by running a 20 µl qPCR reaction (2 µl PCR product, 0.5 µM oligo C [Illumina P5], 0.5 µM oligo D [Ilumina P7], 0.6X SYBR Green I [Thermo Fisher Scientific], and 1X NEBNext High-Fidelity 2X PCR Master Mix [NEB]): 30 s at 98 °C, followed by 20 cycles of 10 s at 98 °C and 30 s at 63 °C and 1 min at 72 °C. The remaining 18 µl PCR product was amplified the number of PCR cycles corresponding to 1/3 of the maximum fluorescence intensity. The amplified PCR product was then purified and concentrated using a Qiagen MinElute column.

### DNA sequencing

All libraries were sequenced on a NextSeq 500 (Illumina) using the high output v2 kit (Illumina) in 76 × 8 × 8 × 76 cycle mode, although 38 bp × 8 × 8 × 38 bp sequencing is sufficient. On average, ~50 K reads were sequenced per cell. Due to the nature of the sequencing libraries 30–40% phiX control v3 (Illumina) was spiked in and 1.5 pM were loaded onto the flow cell.

### Per cell cost estimate

The per cell library preparation cost is conservatively estimated (assuming only 1200 single cells captured per chip) at 81¢/cell: (1) Takara Bio ICELL8 chip (52¢/cell), (2) Illumina Tn5 (24¢/cell), (3) e2Tak polymerase (4¢/cell), (4) other reagents contribute <1% additionally. The additional per cell sequencing cost at the depth used for this report (assuming a 75 cycles NextSeq 500/550 High Output v2 Kit) is approximately 17¢/cell. .

### Data analysis

Illumina sequencing reads in BCL format were demultiplexed by single-cell barcode to fastq files using bcl2fastq (Illumina) according to the manufacturer’s manual. Reads were trimmed using Cutadapt^16^ (parameters: -a Trans2_rc = CTGTCTCTTATACACATCTCCGAGCCCACGAGACA, Trans1_rc = CTGTCTCTTATACACATCTGACG CTGCCGACGA) and aligned to either the human (hg19) or mouse (mm9) genomes using Bowtie2^17^. Mitochondrial reads were removed prior to downstream analysis. PCR duplicates were identified and removed if either the start or end position was shared with another sequencing read. Library complexity estimates were obtained using the Picard Tools MarkDuplicates utility (https://broad-institute.github.io/picard/), except for emtpy well where too few reads were present for a robust estimate; in the latter case, the library complexity was estimated as the number of unique reads observed. Accessible chromatin regions (peaks) were determined using MACS2^18^ (parameters: --format BAMPE --nomodel --call-summits --nolambda --keep-dup all) for mouse embryonic stem cells (mESCs) and human lymphoblastoid (GM12878) cells. A previously published accessible peak set for hematopoiesis was used for PBMC, T- and B-cell analysis^1^. Single cells were selected based on imaging using the supplied ICELL8 CellSelect software (Takara Bio USA). Primary PBMCs with fewer than 500 unique (non-mitochondrial) reads or with <20% (10–15% for mESCs and GM12878 cells) of mappable reads lying within peaks were eliminated from subsequent analysis. Bias-corrected deviations in accessibility near transcription factor motifs were calculated using ChromVar^9^. Bias-corrected deviations were linearly transformed to truncated *z*-scores with minimum and maximum values of −2 and 2, respectively. K-means clustering (*k* = 3) was performed on the 50 most variable transcription factor motifs to assign each single cell to a specific cluster. Transcription factors (rows) were then hierarchically clustered using the ward.D2 agglomeration method^19,20^ within the R pheatmap package^21^, while single cells (columns) were ordered by assigned cluster and cell type (Fig. 2b). Visualizations of clustering and tSNE^10^ analyses were constructed using R scripts.

## Electronic supplementary material

Supplementary Information

## Data Availability

The sequencing data that support the findings of this study are available in Figshare under the following DOIs: Metadata: doi: 10.6084/m9.figshare.7006154.v1; Human monocyte cells: doi: 10.6084/m9.figshare.7005707.v1; Human lymphoblast cells (GM12878): doi: 10.6084/m9.figshare.7005713.v1; Human peripheral blood mononuclear cells (PBMCs): doi: 10.6084/m9.figshare.7005752.v1; Mouse embryonic stem cells (mESCs): doi: 10.6084/m9.figshare.7005710.v1; Human CD8^+^ T cells: doi: 10.6084/m9.figshare.7005701.v1; Human CD4^+^ T cells: doi: 10.6084/m9.figshare.7005698.v1; Human T cells: doi: 10.6084/m9.figshare.7005683.v1; Human B cells: doi: 10.6084/m9.figshare.7005539.v1. All other data are available from the authors upon reasonable request.

## References

[1] Corces MR (2016). Lineage-specific and single-cell chromatin accessibility charts human hematopoiesis and leukemia evolution. Nat. Genet..

[2] Cusanovich DA (2018). The cis-regulatory dynamics of embryonic development at single-cell resolution. Nature.

[3] Buenrostro JD (2015). Single-cell chromatin accessibility reveals principles of regulatory variation. Nature.

[4] Pott S (2017). Simultaneous measurement of chromatin accessibility, DNA methylation, and nucleosome phasing in single cells. eLife.

[5] Jin W (2015). Genome-wide detection of DNase I hypersensitive sites in single cells and FFPE tissue samples. Nature.

[6] Thurman RE (2012). The accessible chromatin landscape of the human genome. Nature.

[7] Cusanovich DA (2015). Multiplex single cell profiling of chromatin accessibility by combinatorial cellular indexing. Science.

[8] Buenrostro JD, Giresi PG, Zaba LC, Chang HY, Greenleaf WJ (2013). Transposition of native chromatin for fast and sensitive epigenomic profiling of open chromatin, DNA-binding proteins and nucleosome position. Nat. Methods.

[9] Schep AN, Wu B, Buenrostro JD, Greenleaf WJ (2017). chromVAR: inferring transcription-factor-associated accessibility from single-cell epigenomic data. Nat. Methods.

[10] van der Maaten L, Hinton G (2008). Visualizing data using t-SNE. J. Mach. Learn. Res..

[11] Chen HM (1995). Neutrophils and monocytes express high levels of PU.1 (Spi-1) but not Spi-B. Blood.

[12] Lloberas J, Soler C, Celada A (1999). The key role of PU.1/SPI-1 in B cells, myeloid cells and macrophages. Immunol. Today.

[13] Di Tullio A (2011). CCAAT/enhancer binding protein alpha (C/EBP(alpha))-induced transdifferentiation of pre-B cells into macrophages involves no overt retrodifferentiation. Proc. Natl Acad. Sci. USA.

[14] Laiosa CV, Stadtfeld M, Xie H, de Andres-Aguayo L, Graf T (2006). Reprogramming of committed T cell progenitors to macrophages and dendritic cells by C/EBP alpha and PU.1 transcription factors. Immunity.

[15] Kohu K (2008). Pleiotropic roles of runx transcription factors in the differentiation and function of T lymphocytes. Curr. Immunol. Rev..

[16] Martin M (2011). Cutadapt removes adapter sequences from high-throughput sequencing reads. EMBnet J..

[17] Langmead B, Salzberg SL (2012). Fast gapped-read alignment with Bowtie 2. Nat. Methods.

[18] Zhang Y (2008). Model-based analysis of ChIP-Seq (MACS). Genome Biol..

[19] Ward JH (1963). Hierarchical grouping to optimize an objective function. J. Am. Stat. Assoc..

[20] Murtagh F, Legendre P (2014). Ward’s hierarchical agglomerative clustering method: which algorithms implement ward’s criterion?. J. Classif..

[21] Kolde, R. *Pheatmap: Pretty Heatmaps*. (R package, version 1.0.8, 2015). Available at: https://CRAN.R-project.org/package=pheatmap.

[22] Xu J (2017). Landscape of monoallelic DNA accessibility in mouse embryonic stem cells and neural progenitor cells. Nat. Genet..

