# Supplementary material for: High-throughput chromatin accessibility profiling at single-cell resolution
Source: Nat Commun. 2018 Sep 7;9:3647. doi: 10.1038/s41467-018-05887-x (PMC6128862; doi:10.1038/s41467-018-05887-x)
Supplement: Supplementary file 1 — Supplementary Information [file 41467_2018_5887_MOESM1_ESM.pdf]

Supplementary Information

High-throughput chromatin accessibility profiling at single-cell resolution

Anja Mezger[1,2*], Sandy Klemm[1,*], Ishminder Mann[3], Kara Brower[4], Alain Mir[3], Magnolia Bostick[3], Andrew Farmer[3], Polly Fordyce[1,4,5,6], Sten Linnarsson[2], and William Greenleaf[1,6,7,#]

[1]Department of Genetics, Stanford University, Stanford, CA, USA.
[2]Department of Medical Biochemistry and Biophysics, Karolinska Institute, Stockholm, Sweden.
[3]Takara Bio USA, Mountain View, CA, USA.
[4]Department of Bioengineering, Stanford University, Stanford, CA, USA.
[5]ChEM-H Institute, Stanford University, Stanford, CA, USA.
[6]Chan Zuckerberg Biohub, San Francisco, CA, USA.
[7]Department of Applied Physics, Stanford University, Stanford, CA, USA.

[*]These authors contributed equally.
[#]To whom correspondence should be addressed: wjg@stanford.edu.

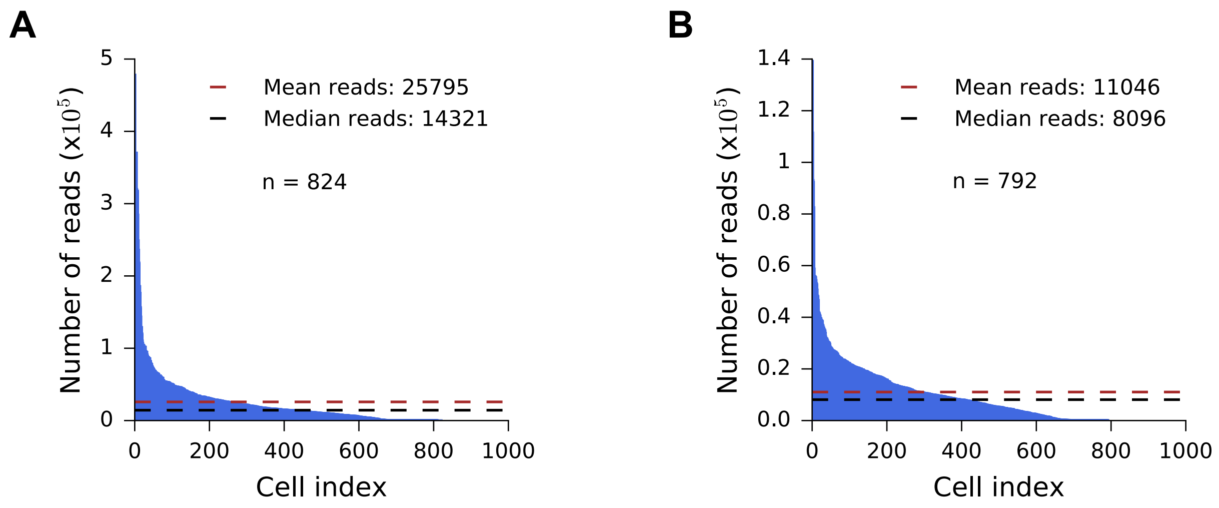

Supplementary Fig. 1 | Number of fragments per cell for (**A**) Human lymphoblastoid GM12878 cells and (**B**) Mouse embryonic stem cells.

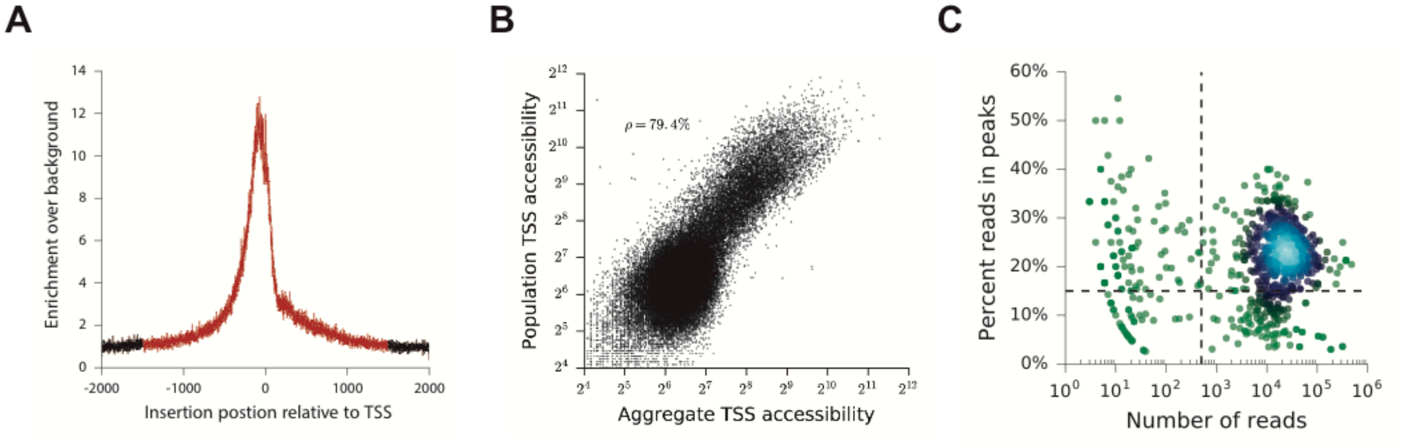

Supplementary Fig. 2 | (**A**) Enrichment of aggregated scATAC-seq fragments at transcription start sites for human lymphoblastoid GM12878 cells. (**B**) Correlation between aggregated scATAC-seq and population ATAC-seq fragments within 2kb upstream and 1kb downstream of transcription start sites for GM12878 cells (2018 Ensembl annotation). (**C**) Signal-to-background (percent reads in peaks) as a function of read depth for human lymphoblastoid GM12828 cells.

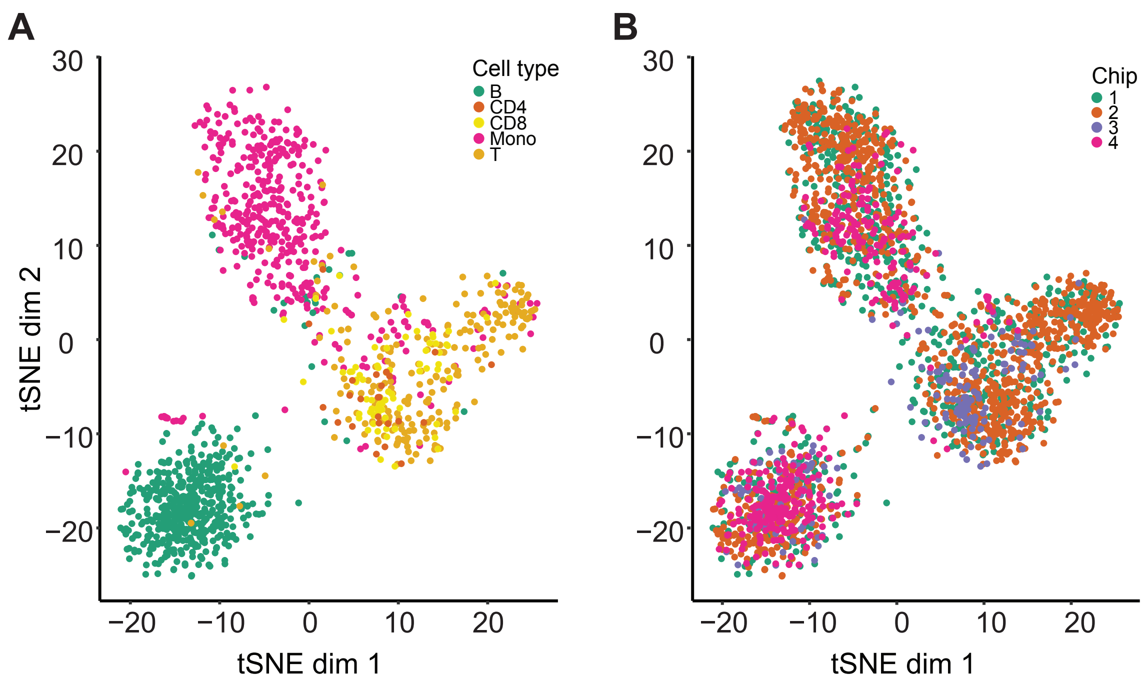

Supplementary Fig. 3 | (**A**) tSNE on normalized ChromVar deviations for $CD4^+$ T, $CD8^+$ T, T, B and monocyte cells labeled by isolated cell type. **(B)** tSNE visualization of all analyzed single cells labeled by chip (note that chip 3 was loaded only with B and T cells and chip 4 was loaded only with monocytes and B cells).

Supplementary Table 1 | The ICELL8 deposition system loads reagents onto a 5184 nano-well chip from a 384 well plate using an 8-channel (2x4) dispense nozzle. The loading chart for the procedure outline in **Figure 1** is shown below: (Step 1, orange, purple, and grey) Sample, fiducial mark and NULL controls; (Step 2, green) Tn5 lysis and transposition reagents; (Step 3, blue) Index 1 with EDTA; (Step 4, pink) Index 2 with $MgCl_2$; (Step 5, yellow) PCR reagents.

	1	2	3	4	5	6	7	8	9	10	11	12	13	14	15	16	17	18	19	20	21	22	23	24
1	Sample 1	Sample 5	Tn5	Tn5	I1.1	I1.17	I1.33	I1.49	I1.65	I1.69	PCR	PCR	I2.1	I2.17	I2.33	I2.49	I2.65	I2.69						NULL
2	Sample 2	Sample 6	Tn5	Tn5	I1.2	I1.18	I1.34	I1.50	I1.66	I1.70	PCR	PCR	I2.2	I2.18	I2.34	I2.50	I2.66	I2.70						
3	Sample 3	Sample 7	Tn5	Tn5	I1.3	I1.19	I1.35	I1.51	I1.67	I1.71	PCR	PCR	I2.3	I2.19	I2.35	I2.51	I2.67	I2.71						
4	Sample 4	Sample 8	Tn5	Tn5	I1.4	I1.20	I1.36	I1.52	I1.68	I1.72	PCR	PCR	I2.4	I2.20	I2.36	I2.52	I2.68	I2.72						
5					I1.5	I1.21	I1.37	I1.53					I2.5	I2.21	I2.37	I2.53								
6					I1.6	I1.22	I1.38	I1.54					I2.6	I2.22	I2.38	I2.54								
7					I1.7	I1.23	I1.39	I1.55					I2.7	I2.23	I2.39	I2.55								
8					I1.8	I1.24	I1.40	I1.56					I2.8	I2.24	I2.40	I2.56								
9					I1.9	I1.25	I1.41	I1.57					I2.9	I2.25	I2.41	I2.57								
10					I1.10	I1.26	I1.42	I1.58					I2.10	I2.26	I2.42	I2.58								
11					I1.11	I1.27	I1.43	I1.59					I2.11	I2.27	I2.43	I2.59								
12					I1.12	I1.28	I1.44	I1.60					I2.12	I2.28	I2.44	I2.60								
13					I1.13	I1.29	I1.45	I1.61					I2.13	I2.29	I2.45	I2.61								
14					I1.14	I1.30	I1.46	I1.62					I2.14	I2.30	I2.46	I2.62								
15					I1.15	I1.31	I1.47	I1.63					I2.15	I2.31	I2.47	I2.63								
16	Fiducial				I1.16	I1.32	I1.48	I1.64					I2.16	I2.32	I2.48	I2.64								NULL

Supplementary Table 2 | Hematopoietic cell type isolation and cluster purity

	T cells	CD4 T cells	CD8 T cells	B cells	Monocytes
**Maximum isolation purity**[1]	97%	96%	91%	95%	92%
**scATAC cluster purity**	91.4%	91.7%	91.7%	95.3%	82.5%

[1]Estimates of isolation purity provided by the manufacture (STEMCELL Technologies Inc.)

Supplementary Table 3 | Custom i5 and i7 indices[1]. All primers have been previously tested and can be used interchangeably.

Custom Adapter 1 (i5):

v2_Ad1.1_TAGATCGC	AATGATACGGCGACCACCGAGATCTACACTAGATCGCTCGTCGGCAGCGTCAGATGTGTAT
v2_Ad1.2_CTCTCTAT	AATGATACGGCGACCACCGAGATCTACACCTCTCTATTCGTCGGCAGCGTCAGATGTGTAT
v2_Ad1.3_TATCCTCT	AATGATACGGCGACCACCGAGATCTACACTATCCTCTTCGTCGGCAGCGTCAGATGTGTAT
v2_Ad1.4_AGAGTAGA	AATGATACGGCGACCACCGAGATCTACACAGAGTAGATCGTCGGCAGCGTCAGATGTGTAT
v2_Ad1.5_GTAAGGAG	AATGATACGGCGACCACCGAGATCTACACGTAAGGAGTCGTCGGCAGCGTCAGATGTGTAT
v2_Ad1.6_ACTGCATA	AATGATACGGCGACCACCGAGATCTACACACTGCATATCGTCGGCAGCGTCAGATGTGTAT
v2_Ad1.7_AAGGAGTA	AATGATACGGCGACCACCGAGATCTACACAAGGAGTATCGTCGGCAGCGTCAGATGTGTAT
v2_Ad1.8_CTAAGCCT	AATGATACGGCGACCACCGAGATCTACACCTAAGCCTTCGTCGGCAGCGTCAGATGTGTAT
v2_Ad1.9_TGGAAATC	AATGATACGGCGACCACCGAGATCTACACTGGAAATCTCGTCGGCAGCGTCAGATGTGTAT
v2_Ad1.10_AACATGAT	AATGATACGGCGACCACCGAGATCTACACAACATGATTCGTCGGCAGCGTCAGATGTGTAT
v2_Ad1.11_TGATGAAA	AATGATACGGCGACCACCGAGATCTACACTGATGAAATCGTCGGCAGCGTCAGATGTGTAT
v2_Ad1.12_GTCGGACT	AATGATACGGCGACCACCGAGATCTACACGTCGGACTTCGTCGGCAGCGTCAGATGTGTAT
v2_Ad1.13_TTTCTAGC	AATGATACGGCGACCACCGAGATCTACACTTTCTAGCTCGTCGGCAGCGTCAGATGTGTAT
v2_Ad1.14_TAACCAAG	AATGATACGGCGACCACCGAGATCTACACTAACCAAGTCGTCGGCAGCGTCAGATGTGTAT
v2_Ad1.15_GTGTATCG	AATGATACGGCGACCACCGAGATCTACACGTGTATCGTCGTCGGCAGCGTCAGATGTGTAT
v2_Ad1.16_TCCATCAA	AATGATACGGCGACCACCGAGATCTACACTCCATCAATCGTCGGCAGCGTCAGATGTGTAT
v2_Ad1.17_TTCGTGCA	AATGATACGGCGACCACCGAGATCTACACTTCGTGCATCGTCGGCAGCGTCAGATGTGTAT
v2_Ad1.18_AGGTTGCC	AATGATACGGCGACCACCGAGATCTACACAGGTTGCCTCGTCGGCAGCGTCAGATGTGTAT
v2_Ad1.19_CCTTATGT	AATGATACGGCGACCACCGAGATCTACACCCTTATGTTCGTCGGCAGCGTCAGATGTGTAT
v2_Ad1.20_CAGCAACG	AATGATACGGCGACCACCGAGATCTACACCAGCAACGTCGTCGGCAGCGTCAGATGTGTAT
v2_Ad1.21_GGTTCAAT	AATGATACGGCGACCACCGAGATCTACACGGTTCAATTCGTCGGCAGCGTCAGATGTGTAT
v2_Ad1.22_ACATTCGT	AATGATACGGCGACCACCGAGATCTACACACATTCGTTCGTCGGCAGCGTCAGATGTGTAT
v2_Ad1.23_GATTCCCA	AATGATACGGCGACCACCGAGATCTACACGATTCCCATCGTCGGCAGCGTCAGATGTGTAT
v2_Ad1.24_CGGACTGC	AATGATACGGCGACCACCGAGATCTACACCGGACTGCTCGTCGGCAGCGTCAGATGTGTAT
v2_Ad1.25_AGCCGTTC	AATGATACGGCGACCACCGAGATCTACACAGCCGTTCTCGTCGGCAGCGTCAGATGTGTAT
v2_Ad1.26_ATTGGGTC	AATGATACGGCGACCACCGAGATCTACACATTGGGTCTCGTCGGCAGCGTCAGATGTGTAT
v2_Ad1.27_TGCATACT	AATGATACGGCGACCACCGAGATCTACACTGCATACTTCGTCGGCAGCGTCAGATGTGTAT
v2_Ad1.28_GGGCTTGG	AATGATACGGCGACCACCGAGATCTACACGGGCTTGGTCGTCGGCAGCGTCAGATGTGTAT
v2_Ad1.29_GACGTGGC	AATGATACGGCGACCACCGAGATCTACACGACGTGGCTCGTCGGCAGCGTCAGATGTGTAT
v2_Ad1.30_GCAAATTT	AATGATACGGCGACCACCGAGATCTACACGCAAATTTTCGTCGGCAGCGTCAGATGTGTAT
v2_Ad1.31_GCAGCCTC	AATGATACGGCGACCACCGAGATCTACACGCAGCCTCTCGTCGGCAGCGTCAGATGTGTAT
v2_Ad1.32_TCCGAGTT	AATGATACGGCGACCACCGAGATCTACACTCCGAGTTTCGTCGGCAGCGTCAGATGTGTAT
v2_Ad1.33_GCATTAAG	AATGATACGGCGACCACCGAGATCTACACGCATTAAGTCGTCGGCAGCGTCAGATGTGTAT
v2_Ad1.34_ACGATAAC	AATGATACGGCGACCACCGAGATCTACACACGATAACTCGTCGGCAGCGTCAGATGTGTAT
v2_Ad1.35_CCTGCGGG	AATGATACGGCGACCACCGAGATCTACACCCTGCGGGTCGTCGGCAGCGTCAGATGTGTAT
v2_Ad1.36_TGATTGTT	AATGATACGGCGACCACCGAGATCTACACTGATTGTTTCGTCGGCAGCGTCAGATGTGTAT
v2_Ad1.37_GGCACGGA	AATGATACGGCGACCACCGAGATCTACACGGCACGGATCGTCGGCAGCGTCAGATGTGTAT
v2_Ad1.38_GATCATTC	AATGATACGGCGACCACCGAGATCTACACGATCATTCTCGTCGGCAGCGTCAGATGTGTAT
v2_Ad1.39_ATGGTCAT	AATGATACGGCGACCACCGAGATCTACACATGGTCATTCGTCGGCAGCGTCAGATGTGTAT
v2_Ad1.40_CGTACCAA	AATGATACGGCGACCACCGAGATCTACACCGTACCAATCGTCGGCAGCGTCAGATGTGTAT
v2_Ad1.41_CCAGTTTA	AATGATACGGCGACCACCGAGATCTACACCCAGTTTATCGTCGGCAGCGTCAGATGTGTAT
v2_Ad1.42_ACCGGCCC	AATGATACGGCGACCACCGAGATCTACACACCGGCCCTCGTCGGCAGCGTCAGATGTGTAT
v2_Ad1.43_CTAGAAGT	AATGATACGGCGACCACCGAGATCTACACCTAGAAGTTCGTCGGCAGCGTCAGATGTGTAT
v2_Ad1.44_CGCCAGAT	AATGATACGGCGACCACCGAGATCTACACCGCCAGATTCGTCGGCAGCGTCAGATGTGTAT
v2_Ad1.45_TCACATGG	AATGATACGGCGACCACCGAGATCTACACTCACATGGTCGTCGGCAGCGTCAGATGTGTAT
v2_Ad1.46_GAACTCGA	AATGATACGGCGACCACCGAGATCTACACGAACTCGATCGTCGGCAGCGTCAGATGTGTAT
v2_Ad1.47_CCACCGTT	AATGATACGGCGACCACCGAGATCTACACCCACCGTTTCGTCGGCAGCGTCAGATGTGTAT

v2_Ad1.48_TAAGTTAC	AATGATACGGCGACCACCGAGATCTACACTAAGTTACTCGTCGGCAGCGTCAGATGTGTAT
v2_Ad1.49_GAGACGTG	AATGATACGGCGACCACCGAGATCTACACGAGACGTGTCGTCGGCAGCGTCAGATGTGTAT
v2_Ad1.50_TTGCCTAA	AATGATACGGCGACCACCGAGATCTACACTTGCCTAATCGTCGGCAGCGTCAGATGTGTAT
v2_Ad1.51_TTAACTTG	AATGATACGGCGACCACCGAGATCTACACTTAACTTGTCGTCGGCAGCGTCAGATGTGTAT
v2_Ad1.52_CTTTAACA	AATGATACGGCGACCACCGAGATCTACACCTTTAACATCGTCGGCAGCGTCAGATGTGTAT
v2_Ad1.53_CGTAGACC	AATGATACGGCGACCACCGAGATCTACACCGTAGACCTCGTCGGCAGCGTCAGATGTGTAT
v2_Ad1.54_TATTTGCG	AATGATACGGCGACCACCGAGATCTACACTATTTGCGTCGTCGGCAGCGTCAGATGTGTAT
v2_Ad1.55_ATCCAGGA	AATGATACGGCGACCACCGAGATCTACACATCCAGGATCGTCGGCAGCGTCAGATGTGTAT
v2_Ad1.56_TGTTCCTG	AATGATACGGCGACCACCGAGATCTACACTGTTCCTGTCGTCGGCAGCGTCAGATGTGTAT
v2_Ad1.57_ACGCGCAG	AATGATACGGCGACCACCGAGATCTACACACGCGCAGTCGTCGGCAGCGTCAGATGTGTAT
v2_Ad1.58_TCTGGCGA	AATGATACGGCGACCACCGAGATCTACACTCTGGCGATCGTCGGCAGCGTCAGATGTGTAT
v2_Ad1.59_AATCTACA	AATGATACGGCGACCACCGAGATCTACACAATCTACATCGTCGGCAGCGTCAGATGTGTAT
v2_Ad1.60_TACTGACC	AATGATACGGCGACCACCGAGATCTACACTACTGACCTCGTCGGCAGCGTCAGATGTGTAT
v2_Ad1.61_CGATAGGG	AATGATACGGCGACCACCGAGATCTACACCGATAGGGTCGTCGGCAGCGTCAGATGTGTAT
v2_Ad1.62_ACTTAGAA	AATGATACGGCGACCACCGAGATCTACACACTTAGAATCGTCGGCAGCGTCAGATGTGTAT
v2_Ad1.63_AGAGATCT	AATGATACGGCGACCACCGAGATCTACACAGAGATCTTCGTCGGCAGCGTCAGATGTGTAT
v2_Ad1.64_GGTGAAGG	AATGATACGGCGACCACCGAGATCTACACGGTGAAGGTCGTCGGCAGCGTCAGATGTGTAT
v2_Ad1.65_ATCGAATG	AATGATACGGCGACCACCGAGATCTACACATCGAATGTCGTCGGCAGCGTCAGATGTGTAT
v2_Ad1.66_TCAAGAGC	AATGATACGGCGACCACCGAGATCTACACTCAAGAGCTCGTCGGCAGCGTCAGATGTGTAT
v2_Ad1.67_GCCCACGT	AATGATACGGCGACCACCGAGATCTACACGCCCACGTTCGTCGGCAGCGTCAGATGTGTAT
v2_Ad1.68_TGGGCGGT	AATGATACGGCGACCACCGAGATCTACACTGGGCGGTTCGTCGGCAGCGTCAGATGTGTAT
v2_Ad1.69_CCCTTGGA	AATGATACGGCGACCACCGAGATCTACACCCCTTGGATCGTCGGCAGCGTCAGATGTGTAT
v2_Ad1.70_ATTACCGT	AATGATACGGCGACCACCGAGATCTACACATTACCGTTCGTCGGCAGCGTCAGATGTGTAT
v2_Ad1.71_AGTCCGAG	AATGATACGGCGACCACCGAGATCTACACAGTCCGAGTCGTCGGCAGCGTCAGATGTGTAT
v2_Ad1.72_ACTTGTTG	AATGATACGGCGACCACCGAGATCTACACACTTGTTGTCGTCGGCAGCGTCAGATGTGTAT
v2_Ad1.73_GTAATACA	AATGATACGGCGACCACCGAGATCTACACGTAATACATCGTCGGCAGCGTCAGATGTGTAT
v2_Ad1.74_GGCGTCTA	AATGATACGGCGACCACCGAGATCTACACGGCGTCTATCGTCGGCAGCGTCAGATGTGTAT
v2_Ad1.75_GCGCTGCT	AATGATACGGCGACCACCGAGATCTACACGCGCTGCTTCGTCGGCAGCGTCAGATGTGTAT
v2_Ad1.76_GTGCCATT	AATGATACGGCGACCACCGAGATCTACACGTGCCATTTCGTCGGCAGCGTCAGATGTGTAT
v2_Ad1.77_TAGGTATG	AATGATACGGCGACCACCGAGATCTACACTAGGTATGTCGTCGGCAGCGTCAGATGTGTAT
v2_Ad1.78_AACACCTA	AATGATACGGCGACCACCGAGATCTACACAACACCTATCGTCGGCAGCGTCAGATGTGTAT
v2_Ad1.79_CTCCGAAC	AATGATACGGCGACCACCGAGATCTACACCTCCGAACTCGTCGGCAGCGTCAGATGTGTAT
v2_Ad1.80_CAACGGCA	AATGATACGGCGACCACCGAGATCTACACCAACGGCATCGTCGGCAGCGTCAGATGTGTAT
v2_Ad1.81_CAATGTAG	AATGATACGGCGACCACCGAGATCTACACCAATGTAGTCGTCGGCAGCGTCAGATGTGTAT
v2_Ad1.82_GGCTACCC	AATGATACGGCGACCACCGAGATCTACACGGCTACCCTCGTCGGCAGCGTCAGATGTGTAT
v2_Ad1.83_AAAGTCCG	AATGATACGGCGACCACCGAGATCTACACAAAGTCCGTCGTCGGCAGCGTCAGATGTGTAT
v2_Ad1.84_TTCCGCGG	AATGATACGGCGACCACCGAGATCTACACTTCCGCGGTCGTCGGCAGCGTCAGATGTGTAT
v2_Ad1.85_AGGCACTT	AATGATACGGCGACCACCGAGATCTACACAGGCACTTTCGTCGGCAGCGTCAGATGTGTAT
v2_Ad1.86_CTTCAGTG	AATGATACGGCGACCACCGAGATCTACACCTTCAGTGTCGTCGGCAGCGTCAGATGTGTAT
v2_Ad1.87_GCCGGTAG	AATGATACGGCGACCACCGAGATCTACACGCCGGTAGTCGTCGGCAGCGTCAGATGTGTAT
v2_Ad1.88_TTCAATCC	AATGATACGGCGACCACCGAGATCTACACTTCAATCCTCGTCGGCAGCGTCAGATGTGTAT
v2_Ad1.89_CCACACAC	AATGATACGGCGACCACCGAGATCTACACCCACACACTCGTCGGCAGCGTCAGATGTGTAT
v2_Ad1.90_ATATTATC	AATGATACGGCGACCACCGAGATCTACACATATTATCTCGTCGGCAGCGTCAGATGTGTAT
v2_Ad1.91_CCGAAGCA	AATGATACGGCGACCACCGAGATCTACACCCGAAGCATCGTCGGCAGCGTCAGATGTGTAT
v2_Ad1.92_GTATCGGT	AATGATACGGCGACCACCGAGATCTACACGTATCGGTTCGTCGGCAGCGTCAGATGTGTAT

Custom Adapter 2 (i7):

v2_Ad2.1_TAAGGCGA	CAAGCAGAAGACGGCATACGAGATTCGCCTTAGTCTCGTGGGCTCGGAGATGTG
v2_Ad2.2_CGTACTAG	CAAGCAGAAGACGGCATACGAGATCTAGTACGGTCTCGTGGGCTCGGAGATGTG
v2_Ad2.3_AGGCAGAA	CAAGCAGAAGACGGCATACGAGATTTCTGCCTGTCTCGTGGGCTCGGAGATGTG
v2_Ad2.4_TCCTGAGC	CAAGCAGAAGACGGCATACGAGATGCTCAGGAGTCTCGTGGGCTCGGAGATGTG

v2_Ad2.5_GGACTCCT	CAAGCAGAAGACGGCATACGAGATAGGAGTCCGTCTCGTGGGCTCGGAGATGTG
v2_Ad2.6_TAGGCATG	CAAGCAGAAGACGGCATACGAGATCATGCCTAGTCTCGTGGGCTCGGAGATGTG
v2_Ad2.7_CTCTCTAC	CAAGCAGAAGACGGCATACGAGATGTAGAGAGGTCTCGTGGGCTCGGAGATGTG
v2_Ad2.8_CAGAGAGG	CAAGCAGAAGACGGCATACGAGATCCTCTCTGGTCTCGTGGGCTCGGAGATGTG
v2_Ad2.9_GCTACGCT	CAAGCAGAAGACGGCATACGAGATAGCGTAGCGTCTCGTGGGCTCGGAGATGTG
v2_Ad2.10_CGAGGCTG	CAAGCAGAAGACGGCATACGAGATCAGCCTCGGTCTCGTGGGCTCGGAGATGTG
v2_Ad2.11_AAGAGGCA	CAAGCAGAAGACGGCATACGAGATTGCCTCTTGTCTCGTGGGCTCGGAGATGTG
v2_Ad2.12_GTAGAGGA	CAAGCAGAAGACGGCATACGAGATTCCTCTACGTCTCGTGGGCTCGGAGATGTG
v2_Ad2.13_TGGATCTG	CAAGCAGAAGACGGCATACGAGATCAGATCCAGTCTCGTGGGCTCGGAGATGTG
v2_Ad2.14_CCGTTTGT	CAAGCAGAAGACGGCATACGAGATACAAACGGGTCTCGTGGGCTCGGAGATGTG
v2_Ad2.15_TGCTGGGT	CAAGCAGAAGACGGCATACGAGATACCCAGCAGTCTCGTGGGCTCGGAGATGTG
v2_Ad2.16_AGGTTGGG	CAAGCAGAAGACGGCATACGAGATCCCAACCTGTCTCGTGGGCTCGGAGATGTG
v2_Ad2.17_GTGTGGTG	CAAGCAGAAGACGGCATACGAGATCACCACACGTCTCGTGGGCTCGGAGATGTG
v2_Ad2.18_TGGGTTTC	CAAGCAGAAGACGGCATACGAGATGAAACCCAGTCTCGTGGGCTCGGAGATGTG
v2_Ad2.19_TGGTCACA	CAAGCAGAAGACGGCATACGAGATTGTGACCAGTCTCGTGGGCTCGGAGATGTG
v2_Ad2.20_TTGACCCT	CAAGCAGAAGACGGCATACGAGATAGGGTCAAGTCTCGTGGGCTCGGAGATGTG
v2_Ad2.21_CGCGGACA	CAAGCAGAAGACGGCATACGAGATTGTCCGCGGTCTCGTGGGCTCGGAGATGTG
v2_Ad2.22_TTCCATAT	CAAGCAGAAGACGGCATACGAGATATATGGAAGTCTCGTGGGCTCGGAGATGTG
v2_Ad2.23_AATTCGTT	CAAGCAGAAGACGGCATACGAGATAACGAATTGTCTCGTGGGCTCGGAGATGTG
v2_Ad2.24_GGCGTCGA	CAAGCAGAAGACGGCATACGAGATTCGACGCCGTCTCGTGGGCTCGGAGATGTG
v2_Ad2.25_ACAAAGTG	CAAGCAGAAGACGGCATACGAGATCACTTTGTGTCTCGTGGGCTCGGAGATGTG
v2_Ad2.26_TACTTGAA	CAAGCAGAAGACGGCATACGAGATTTCAAGTAGTCTCGTGGGCTCGGAGATGTG
v2_Ad2.27_GTGATAGC	CAAGCAGAAGACGGCATACGAGATGCTATCACGTCTCGTGGGCTCGGAGATGTG
v2_Ad2.28_AGTAGATT	CAAGCAGAAGACGGCATACGAGATAATCTACTGTCTCGTGGGCTCGGAGATGTG
v2_Ad2.29_ATTGCCGG	CAAGCAGAAGACGGCATACGAGATCCGGCAATGTCTCGTGGGCTCGGAGATGTG
v2_Ad2.30_TTGCTAAG	CAAGCAGAAGACGGCATACGAGATCTTAGCAAGTCTCGTGGGCTCGGAGATGTG
v2_Ad2.31_ATAAGTTA	CAAGCAGAAGACGGCATACGAGATTAACTTATGTCTCGTGGGCTCGGAGATGTG
v2_Ad2.32_ATCACTCG	CAAGCAGAAGACGGCATACGAGATCGAGTGATGTCTCGTGGGCTCGGAGATGTG
v2_Ad2.33_GTTAACAG	CAAGCAGAAGACGGCATACGAGATCTGTTAACGTCTCGTGGGCTCGGAGATGTG
v2_Ad2.34_AATGGTAG	CAAGCAGAAGACGGCATACGAGATCTACCATTGTCTCGTGGGCTCGGAGATGTG
v2_Ad2.35_GAGCACGT	CAAGCAGAAGACGGCATACGAGATACGTGCTCGTCTCGTGGGCTCGGAGATGTG
v2_Ad2.36_TTTCGTCA	CAAGCAGAAGACGGCATACGAGATTGACGAAAGTCTCGTGGGCTCGGAGATGTG
v2_Ad2.37_CAAGAATT	CAAGCAGAAGACGGCATACGAGATAATTCTTGGTCTCGTGGGCTCGGAGATGTG
v2_Ad2.38_GAAATGCC	CAAGCAGAAGACGGCATACGAGATGGCATTTCGTCTCGTGGGCTCGGAGATGTG
v2_Ad2.39_AACGCCAT	CAAGCAGAAGACGGCATACGAGATATGGCGTTGTCTCGTGGGCTCGGAGATGTG
v2_Ad2.40_CCTCGCAG	CAAGCAGAAGACGGCATACGAGATCTGCGAGGGTCTCGTGGGCTCGGAGATGTG
v2_Ad2.41_TACACCTC	CAAGCAGAAGACGGCATACGAGATGAGGTGTAGTCTCGTGGGCTCGGAGATGTG
v2_Ad2.42_GGTCATTT	CAAGCAGAAGACGGCATACGAGATAAATGACCGTCTCGTGGGCTCGGAGATGTG
v2_Ad2.43_CAATCTTA	CAAGCAGAAGACGGCATACGAGATTAAGATTGGTCTCGTGGGCTCGGAGATGTG
v2_Ad2.44_TGTGCCTT	CAAGCAGAAGACGGCATACGAGATAAGGCACAGTCTCGTGGGCTCGGAGATGTG
v2_Ad2.45_TCTTATTA	CAAGCAGAAGACGGCATACGAGATTAATAAGAGTCTCGTGGGCTCGGAGATGTG
v2_Ad2.46_GACTTAGT	CAAGCAGAAGACGGCATACGAGATACTAAGTCGTCTCGTGGGCTCGGAGATGTG
v2_Ad2.47_AGACCAGC	CAAGCAGAAGACGGCATACGAGATGCTGGTCTGTCTCGTGGGCTCGGAGATGTG
v2_Ad2.48_AAATACAG	CAAGCAGAAGACGGCATACGAGATCTGTATTTGTCTCGTGGGCTCGGAGATGTG
v2_Ad2.49_TTATGAAA	CAAGCAGAAGACGGCATACGAGATTTTCATAAGTCTCGTGGGCTCGGAGATGTG
v2_Ad2.50_CTTGGGTC	CAAGCAGAAGACGGCATACGAGATGACCCAAGGTCTCGTGGGCTCGGAGATGTG
v2_Ad2.51_CCAAATAA	CAAGCAGAAGACGGCATACGAGATTTATTTGGGTCTCGTGGGCTCGGAGATGTG
v2_Ad2.52_GCGTTAAA	CAAGCAGAAGACGGCATACGAGATTTTAACGCGTCTCGTGGGCTCGGAGATGTG
v2_Ad2.53_CATCCTGT	CAAGCAGAAGACGGCATACGAGATACAGGATGGTCTCGTGGGCTCGGAGATGTG
v2_Ad2.54_GGAGTAAG	CAAGCAGAAGACGGCATACGAGATCTTACTCCGTCTCGTGGGCTCGGAGATGTG
v2_Ad2.55_GACGCTCC	CAAGCAGAAGACGGCATACGAGATGGAGCGTCGTCTCGTGGGCTCGGAGATGTG

v2_Ad2.56_TTCGCGGC	CAAGCAGAAGACGGCATACGAGATGCCGCGAAGTCTCGTGGGCTCGGAGATGTG
v2_Ad2.57_CGGTTCCC	CAAGCAGAAGACGGCATACGAGATGGGAACCGGTCTCGTGGGCTCGGAGATGTG
v2_Ad2.58_ACCGGCTA	CAAGCAGAAGACGGCATACGAGATTAGCCGGTGTCTCGTGGGCTCGGAGATGTG
v2_Ad2.59_CTCATGGG	CAAGCAGAAGACGGCATACGAGATCCCATGAGGTCTCGTGGGCTCGGAGATGTG
v2_Ad2.60_TTTAATGC	CAAGCAGAAGACGGCATACGAGATGCATTAAAGTCTCGTGGGCTCGGAGATGTG
v2_Ad2.61_AAACGGTC	CAAGCAGAAGACGGCATACGAGATGACCGTTTGTCTCGTGGGCTCGGAGATGTG
v2_Ad2.62_GATCCAAA	CAAGCAGAAGACGGCATACGAGATTTTGGATCGTCTCGTGGGCTCGGAGATGTG
v2_Ad2.63_ATGATGAT	CAAGCAGAAGACGGCATACGAGATATCATCATGTCTCGTGGGCTCGGAGATGTG
v2_Ad2.64_CCAACACG	CAAGCAGAAGACGGCATACGAGATCGTGTTGGGTCTCGTGGGCTCGGAGATGTG
v2_Ad2.65_TAACAACA	CAAGCAGAAGACGGCATACGAGATTGTTGTTAGTCTCGTGGGCTCGGAGATGTG
v2_Ad2.66_GGTAAACC	CAAGCAGAAGACGGCATACGAGATGGTTTACCGTCTCGTGGGCTCGGAGATGTG
v2_Ad2.67_CATCGACC	CAAGCAGAAGACGGCATACGAGATGGTCGATGGTCTCGTGGGCTCGGAGATGTG
v2_Ad2.68_ATGGGAAC	CAAGCAGAAGACGGCATACGAGATGTTCCCATGTCTCGTGGGCTCGGAGATGTG
v2_Ad2.69_CGGCCAAT	CAAGCAGAAGACGGCATACGAGATATTGGCCGGTCTCGTGGGCTCGGAGATGTG
v2_Ad2.70_GGGAATGA	CAAGCAGAAGACGGCATACGAGATTCATTCCCGTCTCGTGGGCTCGGAGATGTG
v2_Ad2.71_GTATTCGG	CAAGCAGAAGACGGCATACGAGATCCGAATACGTCTCGTGGGCTCGGAGATGTG
v2_Ad2.72_TCAGCTAT	CAAGCAGAAGACGGCATACGAGATATAGCTGAGTCTCGTGGGCTCGGAGATGTG
v2_Ad2.73_ATTTATCT	CAAGCAGAAGACGGCATACGAGATAGATAAATGTCTCGTGGGCTCGGAGATGTG
v2_Ad2.74_ACAGTTGC	CAAGCAGAAGACGGCATACGAGATGCAACTGTGTCTCGTGGGCTCGGAGATGTG
v2_Ad2.75_CCCGAGAT	CAAGCAGAAGACGGCATACGAGATATCTCGGGGTCTCGTGGGCTCGGAGATGTG
v2_Ad2.76_TAATGTCT	CAAGCAGAAGACGGCATACGAGATAGACATTAGTCTCGTGGGCTCGGAGATGTG
v2_Ad2.77_GCCAATTC	CAAGCAGAAGACGGCATACGAGATGAATTGGCGTCTCGTGGGCTCGGAGATGTG
v2_Ad2.78_CGCCGTGC	CAAGCAGAAGACGGCATACGAGATGCACGGCGGTCTCGTGGGCTCGGAGATGTG
v2_Ad2.79_CTGACCGA	CAAGCAGAAGACGGCATACGAGATTCGGTCAGGTCTCGTGGGCTCGGAGATGTG
v2_Ad2.80_CATTTCGA	CAAGCAGAAGACGGCATACGAGATTCGAAATGGTCTCGTGGGCTCGGAGATGTG
v2_Ad2.81_GCTTGCCA	CAAGCAGAAGACGGCATACGAGATTGGCAAGCGTCTCGTGGGCTCGGAGATGTG
v2_Ad2.82_TTCTACCA	CAAGCAGAAGACGGCATACGAGATTGGTAGAAGTCTCGTGGGCTCGGAGATGTG
v2_Ad2.83_ACGTGACG	CAAGCAGAAGACGGCATACGAGATCGTCACGTGTCTCGTGGGCTCGGAGATGTG
v2_Ad2.84_TGTCCGCG	CAAGCAGAAGACGGCATACGAGATCGCGGACAGTCTCGTGGGCTCGGAGATGTG
v2_Ad2.85_TTAAACTT	CAAGCAGAAGACGGCATACGAGATAAGTTTAAGTCTCGTGGGCTCGGAGATGTG
v2_Ad2.86_ACCACAAC	CAAGCAGAAGACGGCATACGAGATGTTGTGGTGTCTCGTGGGCTCGGAGATGTG
v2_Ad2.87_GCCTCTGG	CAAGCAGAAGACGGCATACGAGATCCAGAGGCGTCTCGTGGGCTCGGAGATGTG
v2_Ad2.88_TCGCCCAC	CAAGCAGAAGACGGCATACGAGATGTGGGCGAGTCTCGTGGGCTCGGAGATGTG
v2_Ad2.89_CACTAGGC	CAAGCAGAAGACGGCATACGAGATGCCTAGTGGTCTCGTGGGCTCGGAGATGTG
v2_Ad2.90_TCGAAGCC	CAAGCAGAAGACGGCATACGAGATGGCTTCGAGTCTCGTGGGCTCGGAGATGTG
v2_Ad2.91_GCATGTAC	CAAGCAGAAGACGGCATACGAGATGTACATGCGTCTCGTGGGCTCGGAGATGTG
v2_Ad2.92_GTTCGAGT	CAAGCAGAAGACGGCATACGAGATACTCGAACGTCTCGTGGGCTCGGAGATGTG
v2_Ad2.93_CCGGGCGC	CAAGCAGAAGACGGCATACGAGATGCGCCCGGGTCTCGTGGGCTCGGAGATGTG
v2_Ad2.94_AGATTTAA	CAAGCAGAAGACGGCATACGAGATTTAAATCTGTCTCGTGGGCTCGGAGATGTG
v2_Ad2.95_CACCATTG	CAAGCAGAAGACGGCATACGAGATCAATGGTGGTCTCGTGGGCTCGGAGATGTG
v2_Ad2.96_AATAAGAC	CAAGCAGAAGACGGCATACGAGATGTCTTATTGTCTCGTGGGCTCGGAGATGTG

Supplementary References

1 Buenrostro, J. D. et al. Single-cell chromatin accessibility reveals principles of regulatory variation. *Nature* **523**, 486–490 (2015).
